# The Expression and Prognostic Significance of VEGF and CXCR4 in Gastric Cancer: Correlation with Angiogenesis, Lymphangiogenesis and Progression

**DOI:** 10.3390/cimb44070212

**Published:** 2022-07-06

**Authors:** Łukasz Kruszyna, Dawid Murawa, Paweł Piotr Jagodziński, Grzegorz Oszkinis, Zbigniew Krasiński

**Affiliations:** 1 Department of Vascular and Endovascular Surgery, Angiology and Phlebology, Poznan University of Medical Sciences, Długa ½ St., 61-848 Poznań, Poland; zkrasinski@ump.edu.pl; 2 Department of General Surgery and Surgical Oncology, Faculty of Medicine and Health Sciences, University of Zielona Gora, Zyty 26 St., 65-046 Zielona Góra, Poland; dmurawa@uz.zgora.pl; 3 Department of Biochemistry and Molecular Biology, Poznan University of Medical Sciences, Święcickiego 6 St., 60-781 Poznań, Poland; pjagodzi@ump.edu.pl; 4 Department of Vascular and General Surgery, Institute of Medical Sciences, University of Opole, Witosa 26 St., 45-401 Opole, Poland; grzegorz.oszkinis@uni.opole.pl

**Keywords:** gastric cancer, VEGF, CXCR4, angiogenesis, lymphangiogenesis, lymph node metastases

## Abstract

The cellular response to hypoxia includes the expression of hypoxia-inducible factor-1 (HIF-1) and its target genes: vascular endothelial growth factor (VEGF) and CXC chemokine receptor 4 (CXCR4). The aim of this study was to investigate the expression and prognostic significance of VEGF and CXCR4, which are responsible for angiogenesis and progression in gastric cancer. Twenty-eight gastric cancer patients were analyzed. The mRNA expression was examined in primary tumors and corresponding normal gastric mucosa by RT-PCR. The protein level was examined by immunohistochemistry staining. The high expression of VEGF and CXCR4 was found in 71.0 and 64.0% of tumors, respectively. The mean levels of VEGF and CXCR4 were upregulated in primary tumors compared to normal mucosa (*p* = 0.0007, *p* = 0.0052). A correlation between VEGF expression and tumor invasion (*p* = 0.0216) and stage (*p* = 0.0181) was found. CXCR4 expression correlated with lymph node metastases (*p* = 0.0237) and stage (*p* = 0.0054). The VEGF expression correlated with microvessel density (MVD) (*p* = 0.0491). The overall 3-year survival rate was 46.4% and correlated negatively with high CXCR4 mRNA expression (*p* = 0.0089). VEGF and CXCR4 play an important role in tumor progression. Their overexpression correlates with a bad prognosis and may improve high-risk patient selection, and these patients may obtain additional survival benefits if treated more aggressively.

## 1. Introduction

Gastric cancer is one of the most frequent malignancies, being the third most common reason for cancer-related deaths worldwide [1]. Currently, radical surgical resection remains the only treatment associated with a 5-year survival rate improvement. Despite the benefits of radiotherapy and chemotherapy, the outcome of patients with advanced or metastatic tumors remains poor, with the 5-year survival remaining less than 10% [2]. The prognosis of a patient with gastric cancer has been shown to be influenced by several established clinicopathological characteristics, including tumor size, stage, location, histological type, and grade [3]. Identification of new molecular markers as prognostic and predictive factors and the development of novel agents targeting gastric carcinogenesis are needed.

Regions of hypoxia exist within solid tumors, including gastric cancer. Oxygen can only diffuse 100–180 µm from the blood capillaries to cells [4]. The cellular response to hypoxia includes the expression of hypoxia-inducible factor-1 (HIF-1) and its target genes. A variety of genes, including vascular endothelial growth factor (VEGF) and CXC chemokine receptor 4 (CXCR4), are known to have hypoxia-responsive elements (HRE) and are activated by HIF-1 [5]. These target genes promote angiogenesis, cell proliferation, migration, and metastasis [4,6,7].

Angiogenesis, the process of new vessel development, is one of the critical steps in tumor growth, being involved not only in local extension but also being responsible for metastasis [8]. Angiogenesis is activated early in carcinogenesis due to an imbalance between positive and negative angiogenic factors produced by both tumor cells and normal cells [9]. Among growth factors involved in tumor-related angiogenesis, the VEGF superfamily plays a determinant role [6]. VEGF increases vascular permeability and promotes the formation of new blood vessels by stimulating endothelial cells to migrate and differentiate [10]. A positive correlation between VEGF expression, lymph-node involvement, and lymphatic invasion has been reported in gastric cancer [11]. VEGF has also been reported to function as a vascular permeability factor of tumor vessels responsible for the production of ascites and peritoneal carcinomatosis in gastric cancer [12]. Recently, the inhibition of angiogenesis in gastric cancer has become an area of intensive research [6]. It has been reported that VEGF is an independent and important prognostic factor in human gastric cancer [4,11,13]. On the other hand, there are reports that contradict these results [14]. At present, the most widely used method to assess neovascularization in human malignancies is the quantification of microvessel density (MVD) using specific markers for endothelial cells. Higher MVD scores have been shown to be associated with poor outcomes in gastric cancer [14]. CD34 is an indirect marker of neoangiogenesis and an indicator of MVD. Both VEGF and CD34 are closely correlated with vasculogenesis and angiogenesis in tumor development [13].

Chemokines are a large family of chemotactic cytokines, which, together with their receptors, function as a signaling pathway in leukocyte trafficking and lymphocyte homing [15]. Chemokine receptors are expressed not only by leukocytes but also by certain epithelial cells and several types of cancer cells. Furthermore, these signaling pathways play an essential role in tumor progression. CXCR4 and its chemokine ligand 12 (CXCL12), also known as the stromal cell-derived factor-1 (SDF-1), are two key factors in the cross-talking between tumor cells and their microenvironment [16]. CXCR4 plays a crucial role in the process of neoangiogenesis and migration of metastatic tumor cells [17]. SDF-1 is highly expressed at sites of metastasis, including the lymph nodes and liver. CXCR4 was reported to be expressed in many human tumors and has been found to be a prognostic marker in various types of cancer, including breast cancer and colorectal cancer [18,19]. CXCR4 is one of the genes reported to be positively regulated by HIF-1 [20]. CXCL12 and CXCR4 are expressed at significantly increased levels in gastrointestinal cancers, and this overexpression is associated with the activation of downstream pathways and survival, proliferation, angiogenesis, and migration of tumor cells [16]. Previous studies demonstrated that the CXCL12/CXCR4 axis plays an important role in the metastasis of many malignancies, including gastric cancer. However, a comprehensive analysis of CXCR4 expression concerning the prognosis of patients with gastric cancer remains unknown and needs to be fully established.

The present study was designed to investigate the expression of VEGF, CXCR4 genes, and CD34 (for the assessment of MVD) in gastric cancer tissue using real-time PCR and immunohistochemistry. Furthermore, we investigated whether the expression of VEGF, CXCR4, and MVD has a significant correlation with clinicopathological factors, survival outcomes, and tumor recurrence following the curative resection of gastric cancer.

## 2. Material and Methods

### 2.1. Patients and Tissue Specimens

Twenty-eight patients with gastric cancer were enrolled in this study. Patients were qualified for curative gastrectomy procedures according to the Union for International Cancer Control (UICC). None of the patients had received any preoperative chemotherapy or radiotherapy. During the procedure, a sample of tissue was taken from both cancer and macroscopically normal mucosa at least 5 cm away from the tumor. Additionally, there were 10 normal gastric mucosa samples obtained from patients undergoing gastric procedures for other reasons than cancer. The collected specimens were fresh frozen in liquid nitrogen immediately and stored at −80 °C until RNA extraction. Other specimens were fixed in 10% formalin and embedded in paraffin for pathology examination. The clinicopathological characteristics of these patients were collected and are shown in Table 1. Tumors were staged according to the 7th edition of the tumor-node metastasis (TNM) classification of the UICC [21]. Written informed consent was obtained from all participating individuals. The procedures of the study were approved by the Local Ethical Committee of Poznan University of Medical Sciences.

### 2.2. Reverse Transcription and Real-Time Quantitative Polymerase Chain Reaction (RT-PCR)

The mRNA expression of analyzed genes was examined by reverse transcription-polymerase chain reaction (RT-PCR). The total RNA from cancerous and corresponding normal tissues was isolated according to the method of Chomczynsky and Sacchi [22]. RNA samples were treated with DNase-I (Invitrogen, Life Technologies, Foster City, CA, USA), quantified, and reverse-transcribed into cDNA. RT-PCR was carried out in a LightCyclerTM real-time PCR detection system (Roche Diagnostics, Mannheim, Germany) using SYBR^®^ Green I as a detection dye. The target cDNA was quantified by the relative quantification method using a calibrator. The calibrator was prepared as a cDNA mix from all samples, and successive dilutions were used to create a standard curve as described in the Relative Quantification Manual (Roche Diagnostics). For amplification, 2 µL of cDNA solution was added to 18 µL of QuantiTect^®^ SYBR^®^ Green PCR Master Mix (QIAGEN, Hilden, Germany) and primers (Table 2). The quantity of VEGF and CXCR4 transcript in each sample were standardized by the porphobilinogen deaminase (PBGD) cDNA levels. The VEGF and CXCR4 transcript levels were expressed as a multiplicity of these cDNA concentrations in the calibrator.

### 2.3. Immunohistochemistry

For immunohistochemical staining, we used the Dako EnVisionTM FLEX detection system (DAKO, Carpinteria, CA, USA). A quantity of 4 µm-thick sections were de-paraffinized and rehydrated. The endogenous peroxidase activity was blocked by incubation with 3% hydrogen peroxide for 10 min. The sections were incubated with anti-VEGF (A-20) antibody (Santa Cruz Biotechnology, Santa Cruz, CA, USA) at a dilution of 1:500, and anti-CD34 (43A1) antibody (Santa Cruz Biotechnology) at a dilution of 1:50 for 20 min. For the dilution of concentrated primary antibodies, we used EnVisionTM FLEX Antibody Diluent (DAKO). The sections were washed in TBS for 10 min and incubated for 20 min with Dako EnVisionTM FLEX/HRP (DAKO) detection reagent, which consists of a dextran backbone to which a large number of peroxidase (HRP) molecules and secondary antibody molecules have been coupled. Sections were washed in TBS and incubated with EnVisionTM FLEX DAB+ Chromogen (DAKO) containing 3,3-diaminobenzidine tetrahydrochloride for 5 min according to the manufacturer’s instructions. Finally, slides were counterstained with Mayer hematoxylin and mounted in an aqueous medium. The tissue samples were dehydrated through graded concentrations of ethanol. Negative controls were performed by replacing the specific primary antibody with PBS. The control consisted of a normal stomach tissue obtained from a distant region of the tumor. The positive control consisted of tissue sections of known positive immunoreaction for each antibody.

### 2.4. Histopathological Evaluation

The pathologist who scored the tissue samples was blinded to the clinical data. The immunohistochemical results for VEGF were classified as follows: (-) no staining; (+) staining in less than 10% of cells; (++) staining in 10–50% of cells; (+++) staining in more than 50% of cells. For statistical analysis, tumors having a staining score of negative or low (+) expression were considered negative, whereas tumors with moderate (++) or strong (+++) staining were considered positive.

MVD was assessed by immunohistochemistry using a CD34 marker. We measured MVD by microscopically examination of sections from representative zones with the most significant number of capillaries as described by Weidner N [23]. After examination with a small objective (×50) and selection of zones with increased MVD “hot spots,” we counted the microvessels with a larger objective (×200). Microvessel density represents the average number of vessels counted on five hot spots with an objective of ×200. For statistical analysis, tumors having weak CD34 staining scores (+) were combined into a low-expression group and were compared to tumors with moderate (++) or high (+++) staining (high-expression group).

### 2.5. Statistical Analysis

Continuous variables were tested for normality using the Shapiro–Wilk test. The obtained data were presented as mean ± standard deviation (SD) in a case of a normal distribution or as median and range in other distributions. Categorical variables are presented as counts and percentages.

A two-sided Fisher’s exact test was used to examine the correlation between VEGF and CXCR4 expression and various clinicopathological factors. The significance of differences between mRNA expression levels in cancer, corresponding noncancerous tissue, and control tissue was determined by the nonparametric Wilcoxon or Mann–Whitney U (for independent groups) tests as appropriate. The overall patient survival rate was analyzed with the Kaplan–Meier method. The difference in survival curves was evaluated with a log-rank test. In all tests, a value of *p* < 0.05 was considered statistically significant.

## 3. Results

### 3.1. Patients and Clinicopathological Findings

The characteristics of patients included in the study are shown in Table 1. The study group consisted of 20 men and 8 women. The median age was 64 years (range 21–79 years). The median follow-up period was 27.5 months (range 0.5–58 months).

### 3.2. The Expression of VEGF and CXCR4 mRNA

**Figure 1:**
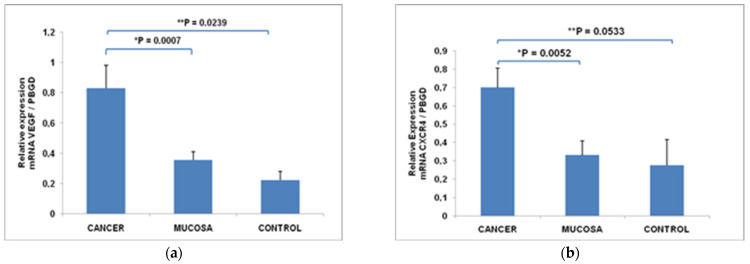
The expression level of VEGF mRNA (**a**) and CXCR4 mRNA (**b**) in primary tumors, corresponding normal gastric mucosa and control tissue. Data are presented as mean ± SEM; * difference in mRNA expression between cancer and corresponding normal mucosa (Wilcoxon test); ** difference in mRNA expression between cancer and control tissue (Mann–Whitney U test).

The mean level of VEGF mRNA was significantly upregulated in primary tumors (0.83 ± 0.15) compared to the corresponding normal gastric mucosa (0.36 ± 0.05), *p* = 0.0007; and control tissue (0.22 ± 0.06), *p* = 0.0239 (Figure 1a). The high expression of VEGF mRNA was found in 20 patients (71.0% of tumors). The mean level of CXCR4 mRNA was significantly upregulated in primary tumors (0.70 ± 0.11) compared to the corresponding normal gastric mucosa (0.33 ± 0.08), *p* = 0.0052 and control tissue (0.28 ± 0.14), *p* = 0.0533 (Figure 1b). The high expression of CXCR4 mRNA was found in 18 patients (64.0% of tumors).

### 3.3. Correlation between Clinicopathological Features and the Expression of VEGF and CXCR4 mRNA in Gastric Cancer Tissue

A statistically significant correlation between the expression of VEGF mRNA and the pTNM stage was noticed (*p* = 0.0441) (Table 3). High VEGF-mRNA expression was found to be present in advanced tumors. CXCR4 mRNA expression correlated both with the pN stage (*p* = 0.0028) and pTNM stage (*p* = 0.0054) (Table 3).

### 3.4. Relationship between Depth of Tumor Invasion (pT), Lymph Nodes (pN), Tumor Stage, and the mRNA Expressions of VEGF and CXCR4

**Figure 2:**
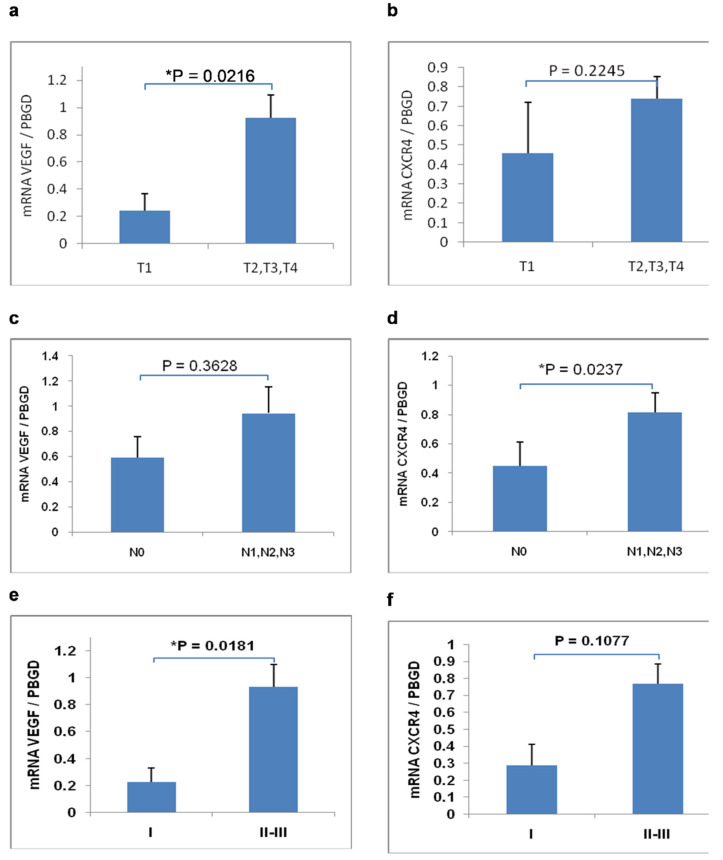
Relationship between mean mRNA expression of VEGF and CXCR4 according to the depth of tumor invasion (pT) (**a**,**b**); lymph node metastases (pN) (**c**,**d**) and stage (**e**,**f**). Data are presented as mean ± SEM; * *p* < 0.05; Mann–Whitney test.

We compared mRNA expression levels in the group of early gastric cancer (pT1) with the group of advanced gastric cancer (pT2-T4). A statistically significant difference was noticed in the mean mRNA expression level of VEGF (Figure 2a). The mean mRNA expression level of VEGF in the pT1 group was significantly lower than in the pT2-T4 group (*p* = 0.0216).

We compared mRNA expression levels in the group of N0 tumors with the group of patients with lymph-node metastases (N1, N2, N3). A statistically significant difference was noticed in the mean mRNA expression level of CXCR4 between the N0 group and the N1–N3 group (Figure 2d). The mean mRNA expression level of CXCR4 in patients with lymph node metastases was significantly higher than in the N0 group (*p* = 0.0237).

We compared mRNA expression levels in different tumor stages. A statistically significant difference was noticed in the mean mRNA expression level of VEGF between stage I and stage II/III (Figure 2e). The mean mRNA expression level of VEGF in stage II/III was significantly higher than in stage I (*p* = 0.0181).

### 3.5. Immunohistochemical Analyses for VEGF and CD34

Immunohistochemical staining for the VEGF and CD34 expressions was successfully performed in 20 patients. VEGF was positive (++/+++) in tumors of 12 patients (60%), whereas it was negative (-/+) in 8 patients (Table 4). The high expression of CD34 marker (++/+++) was found in the tumors of 17 patients (85%), whereas it was low (+) in 3 patients (Table 4).

**Figure 3:**
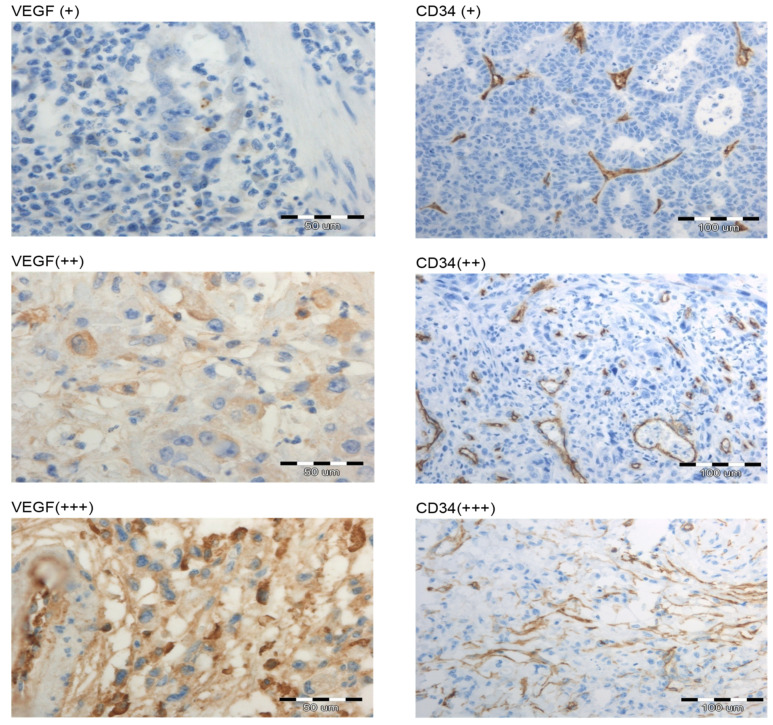
Immunohistological staining for VEGF and CD34 in primary gastric cancer samples. Positive staining was observed as a brown color. Representative examples of low (+), moderate (++), and strong (+++) expression (magnification 200×).

The immunohistological staining for VEGF and CD34 in primary tumors is presented in Figure 3. The VEGF expression was mainly localized in the cytoplasm and membrane of the tumor cells. VEGF and CD34 immunological staining did not correlate significantly with clinicopathological parameters and survival but were strongly associated.

CD34 expression was significantly more frequent in tumors with a high VEGF expression (*p* = 0.0491), as shown in Table 5. 

### 3.6. Correlation between VEGF mRNA Expression and VEGF Immunostaining

The analysis showed a significant correlation between high VEGF-mRNA expression in primary gastric cancer tissue and VEGF immunostaining (*p* = 0.0256). In the group of patients with negative (-/+) VEGF expression, the mean level of mRNA expression was significantly lower than in the group with positive (++/+++) VEGF expression.

### 3.7. The VEGF and CXCR4 Expression and Survival

**Figure 4:**
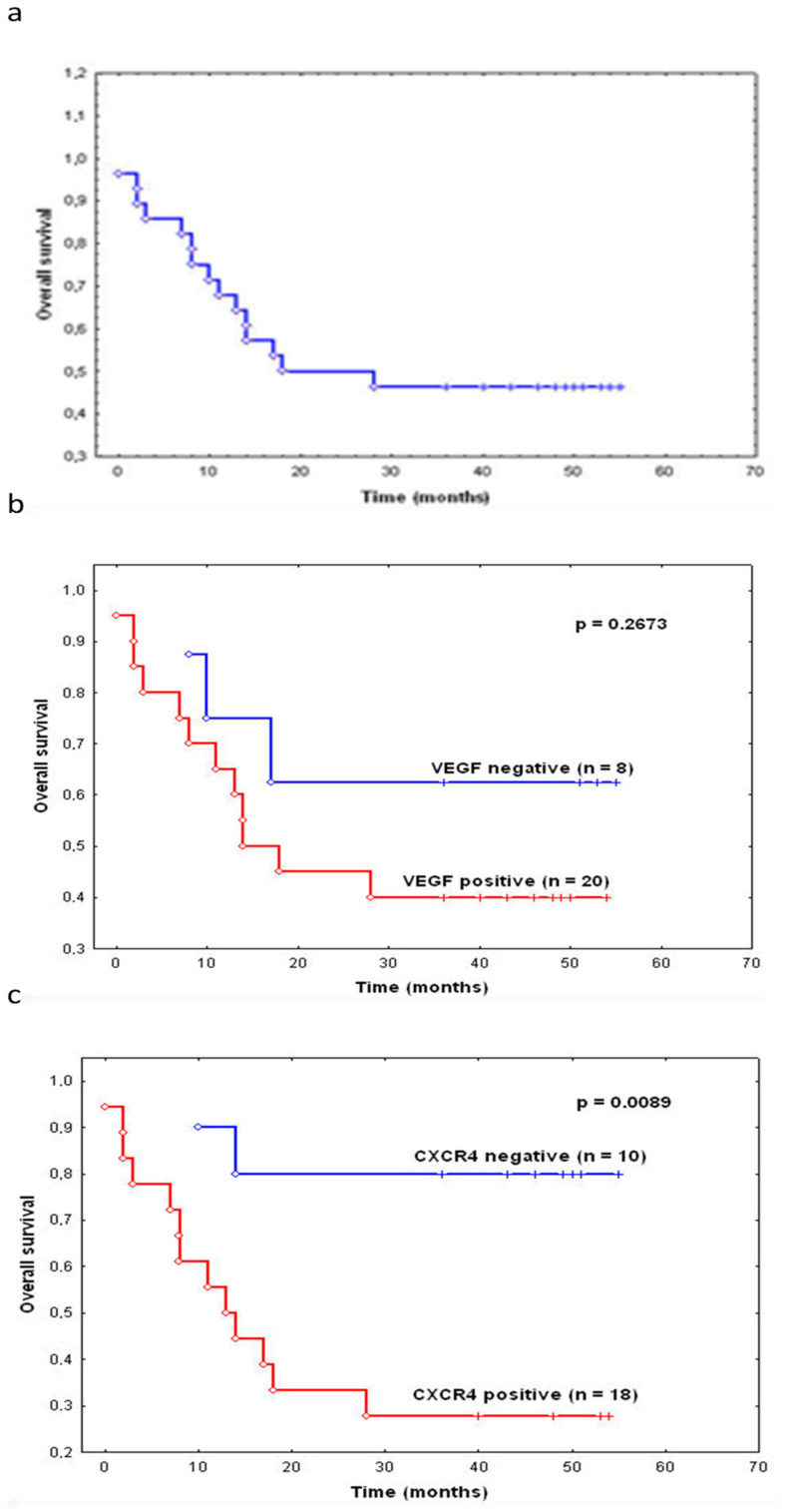
The 3-year overall survival in patients with surgically resected gastric cancer (**a**). The Kaplan–Meier survival curves according to the level of VEGF mRNA (**b**) and CXCR4 (**c**) expression in gastric cancer tissues; log-rank test.

The 3-year overall survival rate was 46.4% (Figure 4a). The analysis of survival curves according to VEGF expression showed the difference between patients with high and low expression in overall survival. Those with low VEGF mRNA expression had a better prognosis compared to the group with high expression (Figure 4b); however, the difference was not statistically significant (*p* = 0.2673). The 3-year survival rate in the patients with high CXCR4 mRNA expression was significantly lower than in those patients with low CXCR4 expression (*p* = 0.0089) (Figure 4).

## 4. Discussion

VEGF is produced by a variety of tumors, including gastric cancer. The data suggest that VEGF expression has a negative influence on prognosis [11,13,24]. A positive correlation between VEGF expression and lymph node involvement and patients’ survival in gastric cancer has been reported [24]. In most studies, VEGF expression is correlated with clinicopathological variables, including tumor size, grading, depth of invasion, lymph-node status, vascular invasion, and distant metastasis. The negative influence of VEGF expression is proven, but the results on the correlation between VEGF and those clinical factors are conflicting and inconclusive [25]. It is unknown whether the differences in these investigations are due to limited sample size or the heterogeneity of gastric cancer among different populations. Several clinical studies support the idea of biological differences in gastric adenocarcinomas arising in Western versus Asian patients. Serum VEGF–A was shown to predict survival in Caucasian but not Asian patients undergoing resection for gastric adenocarcinoma [26]. The existing differences between various studies could also be due to VEGF polymorphisms, which may contribute to gastric tumor characteristics [27]. Although the association between VEGF polymorphisms and gastric cancer risk has been extensively studied, the results available remain controversial [28].

In the presented study, the role of VEGF was assessed according to its influence on the survival rate of patients with gastric cancer. In our study, consistent with previous reports, we found that levels of VEGF in gastric cancer tissue were significantly increased in comparison with the corresponding normal gastric mucosa. Other reports detected a similar VEGF expression rate in gastric cancer tissue. Kolev et al. found that VEGF was expressed in 50.3% of patients [29]. In our study, a positive correlation was observed between VEGF and CD34 expression in gastric carcinoma samples. This result is consistent with previous studies that reported a significant correlation between the expression of VEGF and MVD [14].

In the following part of our study, the results were compared with the tumor stage and survival. It was previously reported that VEGF protein expression in gastric cancer tissues is positively correlated with TNM staging and lymph-node metastasis in patients [11]. A statistically significant correlation was found in our patients between VEGF mRNA and the depth of invasion (pT) and pTNM stage, which collectively contribute to the survival of patients with gastric cancer. Pang et al. reported that the expression of VEGF protein in stage T4 gastric cancer tissue was 10 times higher than in stage Tis [11]. Furthermore, the VEGF expression in patients with stage N3 was shown to be about seven times higher than in patients with stage N0 gastric cancer [11]. In our study, there was no significant correlation between VEGF mRNA and lymph-node metastasis. Various studies examined the relationship between VEGF-protein overexpression with clinical outcome in patients with gastric cancer, but due to conflicting results, the prognostic significance of VEGF remains controversial. For example, He et al. reported that the expression of VEGF and CD34 was not significantly related to sex, age, TNM stage, and 5-year survival [14]. According to the meta-analysis published by Chen et al., VEGF protein, an independent marker of angiogenesis, can predict the 5-year survival [25]. Another meta-analysis of 20 studies that evaluated the correlation between VEGF-A overexpression and survival suggested that VEGF-A overexpression had an unfavorable impact on overall survival and disease-free survival in patients with gastric cancer [30]. In our study, the survival rate of patients with high levels of VEGF in gastric cancer tissue was lower than that of patients with low VEGF levels, but it was not statistically significant.

Several limitations of the current studies on VEGF expression in different cancers should be discussed. Most studies use immunohistochemistry to assess VEGF expression status. The cut-off value for VEGF positivity varies from 5 to 50% [25]. Immunohistochemistry is the most practical method for evaluating protein expression, not only providing a semiquantitative assessment of the abundance of proteins but also defining the cellular localization of their expression. However, according to the literature, the VEGF expression rate (assessed by immunohistochemistry) varies from 16.7 to 90.1% of gastric cancers [31]. This bias may result from different experimental methodologies.

The influence of markers of angiogenesis in gastric cancer is currently under debate. The expression of VEGF has been evaluated by studies of the protein levels in most human tumors. However, only a few reports have examined VEGF mRNA in human gastric carcinoma. Little is known about the correlation between the mRNA level and the protein expression of VEGF. In our study, overexpression of VEGF has been confirmed both at the protein and the mRNA levels. The results of VEGF mRNA correlated with the VEGF-protein expression that was assessed by immunohistochemistry. Currently, there are no predictive biomarkers available to predict the benefit of anti-angiogenic agents in the treatment of gastric cancer [6]. Therefore, it is difficult to identify patients with tumors more sensitive to angiogenesis inhibitors.

CXCR4 was reported to be expressed in many human tumors, such as breast and colorectal cancer [19]. However, the role of CXCR4 expression in cancer metastasis is still controversial. In gastric cancer, several studies were able to show the upregulation of CXCR4 in cancer tissue either by IHC or RT-PCR compared to normal gastric tissues [32,33,34,35,36]. Positivity rates for CXCR4 at the primary gastric cancer lesions reach 32.3–80.0% by IHC detection, which are significantly higher than those in the adjacent normal mucosa tissues [32,33,35,37,38,39,40]. The clinical significance of CXCR4 expression in gastric cancer has not been clarified and is still controversial. Ying et al. examined the level of CXCR4 expression by immunohistochemical staining in primary gastric tumor tissues, and positive CXCR4 expression was positively associated with lymph-node metastasis, TNM staging, and disease prognosis [35]. The intensity of CXCR4 in primary gastric cancer lesions was positively associated with TNM staging, LN involvement, and the recurrence/metastasis rate after radical surgery, but negatively with overall survival (OS) and disease-free survival (DFS) [35,36,37,41,42].

In this study, we found a statistically significant difference in the mean mRNA expression level of CXCR4 between cancer tissue and corresponding normal gastric mucosa. The high expression of CXCR4 was significantly correlated with pTNM stage and lymph node status. The results of our study are similar to the data published by Satomura et al., who showed the positive expression of CXCR4 in 59.8% of cases, which was associated with the depth of invasion and stage [42].

There are some controversies about the relationships between CXCR4 expression, Lauren classification, and the differentiation of gastric cancer. He et al. reported that diffuse-type gastric cancer presented higher CXCR4 levels than intestinal-type gastric cancer [36]. In our study, the expression of CXCR4 did not correlate with tumor size, location, grade, or Lauren classification. In the study by He et al., there was no difference in primary tumor location or TNM classification between the high and the low CXCR4 expression group. CXCR4 expression was only associated with Lauren classification [36]. Zhao et al. demonstrated that CXCR4 expression was related to the poor differentiation of cancer cells [38]. It was previously reported that metastasis was associated with the CXCL12/CXCR4 axis in many tumors, including gastric cancer [35]. The CXCL12/CXCR4 axis mediates the directional migration of CXCR4-positive tumor cells to CXCL12-expressing organs such as LNs and the liver [34,38]. Our results show that high expression of the CXCR4 molecule is predictive of lymph-node involvement. This result is in agreement with the findings of previous studies where the expression of CXCR4 was associated with lymph-node metastasis and was shown to play a role with respect to the prediction of lymph-node status, including micrometastasis [33]. However, in the study by Rubi et al., CXCR4 mRNA expression in gastric cancer tissue showed no significant differences between patients with or without lymph and vein infiltration [34]. Other reports have demonstrated that CXCR4 expression was not associated with lymphatic invasion and peritoneal metastasis, suggesting that the role of CXCR4 in gastric cancer is still not defined. [32,39] The prognosis of CXCR4-negative cancer was found to be better than that of CXCR4-positive cancer in the current study. This result is contrary to Kwak et al., who found no association between CXCR4 expression and prognosis [32]. However, other studies suggested that the upregulated expression of CXCR4 is an independent prognostic factor for overall survival following gastrectomy of patients with gastric cancer [35,36,42]. Even though, in our study, CXCR4 expression was not correlated with the depth of tumor invasion (pT), its expression rate was higher in advanced TNM-staged cancers. Similar to a previously published study by Nikzaban et al., we also observed that expression of CXCR4 is significantly higher in stages III and IV than in stages I and II, indicating that CXCR4 could be used as a new factor for gastric cancer staging [41]. It is worth noting that Tsuboi et al. reported no significant correlations between CXCL12 and CXCR4 expressions with peritoneal metastasis or survival in pathological T3-stage gastric cancer patients [39]. Our results may have been influenced by the large percentage of samples that were classified as stage III. These findings indicated a close relationship between CXCR4 expression and gastric cancer progression. The assessment of CXCR4 expression in cancer tissue before surgical resection could give valuable information about the risk of potential lymph-node metastases, including lymph-node micrometastasis (LNMM). At the time of diagnosis of gastric cancer, some patients have already developed LNMM, which is usually not detectable by current diagnostic methods. To date, only a few reports have revealed the relationship between LNMM and CXCR4 expression in gastric cancer [33]. According to the authors, CXCR4 expression predicts lymph-node status, including micrometastasis in gastric cancer. While, in most previous studies, the expression levels of CXCR4 were examined by immunohistochemical staining in primary gastric tumor tissues, the present study intended to investigate the expression profile of CXCR4 on the mRNA level.

Although VEGF and CXCR4 have been characterized individually, little is known about the co-expression of these factors in human gastric cancer. Therefore, we analyzed the expression of both molecules. One limitation of our study is that we did not compare the immunohistochemical results of CXCR4 with the mRNA expression levels. SDF-1 and VEGF have been reported to promote the directional migration and invasion of human cancer cells. Tkacz et al. have found that the serum from gastric cancer patients significantly increased the chemotactic response of CRL-1739 gastric cancer cells [43]. The authors emphasized that the observed significant chemotactic, adhesive, and proliferative serum potential is an additional effect of SDF-1, HGF, VEGF and also other substances participating in the regulation of the metastasis process [43].

Our results regarding the prognostic role of VEGF and CXCR4 expression in gastric cancer must be interpreted with caution due to the small sample size. Because of the small number of cancer specimens, the portion of early gastric cancer and N0 tumors was relatively small in our study. Studies with larger patient groups and longer follow-ups must be performed to confirm the prognostic and predictive value of these molecules in gastric cancer.

The results of the presented research suggest a significant role of VEGF and CXCR4 in the biology of gastric cancer. The possible predictive value of both proteins may be promising. Nevertheless, further analyses are required to assess their usefulness in clinical practice. Identifying specific biomarkers for the metastatic potential of primary gastric cancer would allow better patient selection for more radical treatment.

## Figures and Tables

**Table 1:** Clinicopathological characteristics of gastric cancer patients.

Clinicopathological Factor	N	%
Age (years)		
<60	9	32.0
60–69	10	36.0
≥70	9	32.0
Mean ± SD	62.5 ± 12.7	
Median (Range)	64 (21–79)	
Gender		
Male	20	71.4
Female	8	28.6
Type of resection		
total gastrectomy	20	71.4
subtotal gastrectomy	8	28.6
Lymphadenectomy		
D1	12	42.9
D2	16	57.1
Tumor location		
upper	7	25
middle	5	17.8
lower	15	53.6
whole stomach	1	3.6
Tumor size (cm)		
0.0–3.9	9	32.2
4.0–7.9	10	35.7
8.0–11.9	6	21.4
≥12	3	10.7
Lauren classification		
intestinal	12	42.9
diffuse	7	25
mixed	9	32.1
Grading		
G1	1	3.6
G2	9	32.1
G3	18	64.3
pT stage ^a^		
pT1	4	14.3
pT1a	1	
pT1b	3	
pT2	3	10.7
pT3	11	39.3
pT4	10	35.7
pT4a	7	
pT4b	3	
pN stage ^a^		
pN0	9	32.1
pN1	4	14.3
pN2	4	14.3
pN3	11	39.3
pN3a	5	
pN3b	6	
pTNM stage ^a^		
I	4	14.3
IA	3	
IB	1	
II	8	28.6
IIA	5	
IIB	3	
III	16	57.1
IIIA	4	
IIIB	6	
IIIC	6	
IV	0	0

pT—pathological tumor; pN—pathological lymph node; pTNM—pathological tumor-node metastasis; ^a^—according to the 7th edition of the tumor-node metastasis classification of the Union for International Cancer Control (UICC) [21].

**Table 2:** Primer sequences used for RQ-PCR analysis.

Gene	ENST Number	Starter F (*Forward*) and R (*Reverse*)	Product Size (bp)
*VEGF*	00000372055	F: 5′ GTCACACATCTTCCATCTCC 3′	186
		R: 5′ GTGTGCCCCTGATGCGATG 3′	
*CXCR4*	00000241393	F: 5′ TTCTTAACTGGCATTGTGGG 3′	130
		R: 5′ GAAGCGTGATGACAAAGAGG 3′	
*PBGD*	00000278715	F: 5′ GCCAAGGACCAGGACATC 3′	160
		R: 5′ TCAGGTACAGTTGCCCATC 3′	

**Table 3:** Relationship between VEGF and CXCR4 mRNA expressions and clinicopathologic features.

Clinicopathological	VEGF				CXCR4		
Features							
		-	+	*p*-Value	-	+	*p*-Value
	N	8	20		10	18	
Age (years)				1.0000			0.0113
≤60	10	3	7		7	3	
>60	18	5	13		3	15	
Gender				1.0000			0.6692
F	8	2	6		2	6	
M	20	6	14		8	12	
Tumor size				0.1998			1.0000
≤4 cm	11	5	6		4	7	
>4 cm	17	3	14		6	11	
Lauren classification				0.3828			0.3771
intestinal	12	2	10		4	8	
diffuse	7	2	5		4	3	
mixed	9	4	5		2	7	
pT stage				0.1423			0.6744
T1-T2	7	4	3		3	4	
T3-T4	21	4	17		7	14	
pN stage				0.3715			**0.0028**
N0	9	4	5		7	2	
N1-N3	19	4	15		3	16	
pTNM				**0.0441**			**0.0054**
I-II	12	6	6		8	4	
III-IV	16	2	14		2	14	
Grade				1.0000			0.2474
G1/G2	10	3	7		2	8	
G3	18	5	13		8	10	
Tumor location				0.6618			0.0953
upper	7	1	6		1	6	
middle	5	1	4		4	1	
lower	15	6	9		5	10	
whole stomach	1	0	1		0	1	
Vascular invasion				0.2144			0.098
negative	19	7	12		9	10	
positive	9	1	8		1	8	

(-)—low expression; (+)—high expression; Fisher exact test. Bold values indicate statistically significance.

**Table 4:** Immunohistochemical expression of VEGF and CD34 in primary gastric cancer tissue of 20 patients.

	*n* (20)	%
VEGF		
+ (<10%)	8	40
++ (10–50%)	7	35
+++ (>50%)	5	25
CD34		
+	3	15
++	7	35
+++	10	50

**Table 5:** Relationship between expression level of VEGF and CD34 marker in primary gastric cancer tissue.

	VEGF		
CD34	+ (*n* = 8)	++/+++ (*n* = 12)	* *p*-Value
+ (*n* = 3)	3	0	0.0491
++/+++ (*n* = 17)	5	12	

* Fisher’s exact test.

## Data Availability

Not applicable.
